# Electricity consumption of anesthesia workstations and potential emission savings by avoiding standby

**DOI:** 10.1007/s00101-024-01388-3

**Published:** 2024-02-13

**Authors:** Hendrik Drinhaus, Jorrit Drinhaus, Christine Schumacher, Michael J. Schramm, Wolfgang A. Wetsch

**Affiliations:** 1 Faculty of Medicine and University Hospital of Cologne, Department of Anesthesiology and Intensive Care Medicine, University of Cologne, Kerpener Str. 62, 50937 Cologne, Germany; 2 50937 Cologne, Germany

**Keywords:** Anesthesia workstation, Carbon footprint, Climate change, Cost analysis, Electricity consumption, Energy saving, Electricity cost, Anästhesie-Workstation, CO_2_-Footprint, Klimawandel, Kostenanalyse, Stromverbrauch, Energieeinsparung, Stromkosten

## Abstract

**Background:**

Anesthesiology has a relevant carbon footprint, mainly due to volatile anesthetics (scope 1 emissions). Additionally, energy used in the operating theater (scope 2 emissions) contributes to anesthesia-related greenhouse gas (GHG) emissions.

**Objectives:**

Optimizing the electricity use of medical devices might reduce both GHG emissions and costs might hold potential to reduce anaesthesia-related GHG-emissions and costs. We analyzed the electricity consumption of six different anesthesia workstations, calculated their GHG emissions and electricity costs and investigated the potential to reduce emissions and cost by using the devices in a more efficient way.

**Methods:**

Power consumption (active power in watt , W) was measured with the devices off, in standby mode, or fully on with the measuring instrument SecuLife ST. Devices studied were: Dräger Primus, Löwenstein Medical LeonPlus, Getinge Flow C, Getinge Flow E, GE Carestation 750 and GE Aisys. Calculations of GHG emissions were made with different emission factors, ranging from very low (0.09 kg CO_2_-equivalent/kWh) to very high (0.660 kg CO_2_-equivalent/kWh). Calculations of electricity cost were made assuming a price of 0.25 € per kWh.

**Results:**

Power consumption during operation varied from 58 W (GE CareStation 750) to 136 W (Dräger Primus). In standby, the devices consumed between 88% and 93% of the electricity needed during use. The annual electricity consumption to run 96 devices in a large clinical department ranges between 45 and 105 Megawatt-hours (MWh) when the devices are left in standby during off hours. If 80% of the devices are switched off during off hours, between 20 and 46 MWh can be saved per year in a single institution. At the average emission factor of our hospital, this electricity saving corresponds to a reduction of GHG emissions between 8.5 and 19.8 tons CO_2_-equivalent. At the assumed prices, a cost reduction between 5000 € and 11,600 € could be achieved by this intervention.

**Conclusion:**

The power consumption varies considerably between the different types of anesthesia workstations. All devices exhibit a high electricity consumption in standby mode. Avoiding standby mode during off hours can save energy and thus GHG emissions and cost. The reductions in GHG emissions and electricity cost that can be achieved with this intervention in a large anesthesiology department are modest. Compared with GHG emissions generated by volatile anesthetics, particularly desflurane, optimization of electricity consumption of anesthesia workstations holds a much smaller potential to reduce the carbon footprint of anesthesia; however, as switching off anesthesia workstations overnight is relatively effortless, this behavioral change should be encouraged from both an ecological and economical point of view.

## Abstract (de)

**Hintergrund:**

Der anästhesiologische Betrieb verursacht einen relevanten „carbon footprint“, überwiegend durch volatile Anästhetika (sog. *Scope-1-Emissionen*), aber auch durch den Verbrauch von Energie (sog. *Scope-2-Emissionen*).

**Zielsetzung:**

Eine Optimierung des Energieverbrauchs medizinischer Geräte könnte die Emission von Treibhausgasemissionen sowie Kosten senken. Wir analysierten den Energieverbrauch von sechs Typen von Narkosegeräten und untersuchten Potenziale zur Reduktion von Emissionen und Stromkosten

**Methoden:**

Der Energieverbrauch (Wirkleistung) in Watt (W) wurde mit dem Messgerät Gossen SecuLifeST in den Betriebszuständen Aus, Standby und An gemessen. Die untersuchten Geräte waren: Dräger Primus, Löwenstein Medical LeonPlus, Getinge Flow C, Getinge Flow E, GE Carestation 750, GE Aisys. Welche Treibhausgasemissionen mit dem jeweiligen Stromverbrauch verbunden sind bzw. bei Senkung des Stromverbrauchs eingespart werden könnten, ist vom Emissionsfaktor am jeweiligen Standort (an unserer Klinik 0,427 kg/kWh) abhängig. Die Berechnungen der Treibhausgasemissionen wurden mit verschiedenen Emissionsfaktoren vorgenommen, die sich zwischen 0,07 (niedrigster Wert in der EU) und 0,660 kg (höchster Wert in der EU) CO_2_-Äquivalent pro Kilowattstunde (kWh) bewegten. Bei der Berechnung der Stromkosten wurde ein Preis von 0,25 € pro kWh angenommen.

**Ergebnisse:**

Die Wirkleistung der Gerätetypen im Betrieb variierte zwischen 58 (GE CareStation 750) und 136 W (Dräger Primus). Im Standby verbrauchten die Geräte zwischen 88 und 93 % der Elektrizität, die sie im Vollbetrieb benötigten. Der jährliche Energiebedarf, um die 96 Geräte in unserer anästhesiologischen Klinik zu betreiben, liegt zwischen 45 und 105 Megawattstunden (MWh), wenn die Geräte außerhalb der regulären Betriebszeiten im Standby belassen werden. Die Gesamtkosten für diese Mengen an Elektrizität belaufen sich auf 11.296 bis 26.199 €. Wenn ca. 80 % (76 von 96) der Geräte während dieser Zeiten komplett ausgeschaltet würden, könnten in unserer Klinik jährlich zwischen 20 und 46 MWh eingespart werden. Bei dem aktuellen Emissionsfaktor des in unserem Klinikum genutzten Stroms würde dies eine jährliche Reduktion der Treibhausgasemissionen von 8,5 bis 19,8 t CO2-Äquivalent und eine Kosteneinsparung von 5000 bis 11.600 € bedeuten.

**Schlussfolgerung:**

Der Energieverbrauch schwankt deutlich zwischen den verschiedenen Typen von Narkosegeräten. Alle Gerätetypen weisen einen sehr hohen Energieverbrauch im Standby auf, der demjenigen im Betrieb kaum nachsteht. Die Vermeidung von Standby außerhalb der Betriebszeiten kann Energieverbrauch, Treibhausgasemissionen und Kosten reduzieren. Im Vergleich zu den Treibhausgasemissionen durch volatile Anästhetika, vor allem Desfluran, hat die Optimierung des Energieverbrauchs jedoch ein geringeres Potenzial, den anästhesieassoziierten CO_2_-Fußabdruck zu senken. Da das Ausschalten eines großen Teils der vorgehaltenen Narkosegeräte über Nacht allerdings relativ unaufwändig ist, sollte es aus ökologischen und ökonomischen Gründen durchgeführt werden. Zudem sollten die Herstellerfirmen Standby-Modi entwickeln, die einen relevant niedrigeren Stromverbrauch aufweisen als im Betrieb.

## Treten Sie in den Austausch

Diese Arbeit wurde für *Die Anaesthesiologie* in Englisch eingereicht und angenommen. Die deutsche Zusammenfassung wurde daher etwas ausführlicher gestaltet. Wenn Sie über diese Zusammenfassung hinaus Fragen haben und mehr wissen wollen, nehmen Sie gern in Deutsch über die Korrespondenzadresse am Ende des Beitrags Kontakt auf. Die Autorinnen und Autoren freuen sich auf den Austausch mit Ihnen.

The climate crisis imposes enormous challenges in all aspects of life and has a profound impact on global health [1]. Apart from being affected by climate change, healthcare systems are also major contributors to climate change by emitting greenhouse gases (GHG) [2].

Within hospitals anesthesiology is an area of particularly high emission [3, 4]. This is to a large part due to the greenhouse effect of volatile anesthetics, mainly desflurane [5] but aspects such as plastic waste, electricity consumption or air conditioning also contribute to anesthesia’s carbon footprint [3, 4]. Strategies of “avoid, reduce, reuse, recycle, reprocess” or “reduce, reuse, recycle, rethink and research” have been proposed to reduce waste and other forms of emissions generated by anesthesia [4, 5]. The World Resources Institute defines three scopes of greenhouse gas (GHG) emissions [6]. Scope 1 are direct emissions of GHG: in the context of anesthesia this would be, e.g., desflurane or nitrous oxide. Scope 2 are indirect GHG emission due to energy use, e.g., electricity consumption by medical devices, air conditioning, etc. Scope 3 are other indirect GHG emissions, e.g., GHG emissions resulting from production and transportation of materials used in the operating theater [3, 6, 7].

Scope 1 has been addressed by campaigns to avoid desflurane, reduce the use of other volatile anesthetics by reducing fresh gas flow, or switching to total intravenous anesthesia. Scope 2 experiences less visibility in the anesthesiology community. The influence of individual hospital staff to reduce most sources of scope 2 emissions generated by electricity use is limited. This includes the source of energy used or the efficiency of thermal insulation of hospital buildings. Other sources of scope 2 emissions are within the reach of potential reductions by behavioral changes of the staff. This includes switching off medical devices or lights and reducing air conditioning and ventilation when an operating theater is not in use. It has been shown that scope 1 emissions in anesthesiology largely depend on individual behavior and can be reduced by educational campaigns and similar measures [8–11]. Several groups have demonstrated that medical devices in anesthesia and critical care medicine consume considerable amounts of energy when left in standby and are not switched off completely [12–14].

In this study, we aimed to provide a comprehensive analysis of the electricity consumption of different anesthesia workstations and to calculate the potential savings of electricity, GHG emissions and costs by switching off devices instead of leaving them in standby mode when not in use.

## Methods

An ethical approval was not deemed necessary by the managing director of the ethics committee at the medical faculty of the University of Cologne (managing director: Dr. Guido Grass, chairperson: Prof. Dr. Raymond Voltz). The SQUIRE 2.0 guidelines were adhered to while preparing the manuscript [15].

### Anesthesia workstations

We investigated the following anesthesia workstations:

- Dräger Primus (Drägerwerk AG & Co. KGaA, Lübeck, Germany), software version 4.53.00, year of production 2007.
- Löwenstein Leonplus (Löwenstein Medical SE & Co KG, Bad Ems, Germany), software version 3.5.21, year of production 2014.
- Getinge Flow C (Getinge AB, Göteborg, Sweden), software version 4.10.00, year of production 2021.
- Getinge Flow E (Getinge AB, Göteborg, Sweden), software version 4.10.00, year of production 2021.
- General Electric (GE) Care Station 750 (GE Healthcare, Chicago, IL, USA), software version 10 SP06, year of production 2022.
- General Electric (GE) Aisys (GE Healthcare, Chicago, IL, USA), software version 12 SP00, year of production 2023.

### Measurements

Measurements were performed with the measuring instrument SecuLife ST (Gossen Metrawatt GmbH, Nürnberg, Germany), a multifunctional instrument for testing of electrical medical devices complying with the standards DIN VDE 0751/IEC 62353 and EN 60601. Measurements were performed with fully precharged batteries over periods of 2min, with the anesthesia workstation either switched off, in standby (ready to use), or operating in a steady state with a test lung with tidal volume 600 ml, frequency 12 * min^−1^, positive end expiratory pressure (PEEP) 5 cmH_2_O and fresh gas flow 1 l * min^−1^. Power consumption was expressed as active power in watts (W).

The subsequent calculations were performed with Microsoft Excel (Microsoft Corporation, Redmond, WA, USA), visualizations were created with GraphPad Prism 10.0 (GraphPad Software, Boston, MA, USA).

### Calculations

Reduction of electricity consumption per hour and device by avoiding standby was calculated by subtracting power while switched off from power in standby.

Reduction of GHG emission per hour and device by avoiding standby was calculated by multiplying reduction of electricity consumption (described above) by an emission factor. The emission factor describes the amount of carbon dioxide (CO_2_) or CO_2_-equivalent (CO_2_-eq) released by the generation of an amount (usually 1 Kilowatt-hour [kWh]) of electric energy. The respective latest national emission factors were obtained from the European Energy Agency, the Government of the United Kingdom (UK), and the United States (US) Energy Information Administration [16–19]. The emission factor in our hospital was obtained through the technical subsidiary of our hospital, medfacilities GmbH, from the regional energy provider, RheinEnergie AG. The annual electricity consumption for use of the anesthesia workstations was calculated by multiplying the electricity consumption per hour by the number of hours of operation or standby mode per year. To calculate the annual power consumption and the potential for electricity savings by switching off devices instead of leaving them in standby, the number of workstations in our department was multiplied with the power consumption of either all machines fully on during regular operation hours (50 h per week) and in standby during off hours (118 h per week) or all machines fully on during regular operation and only an indispensable part to serve time-critical emergencies in standby and the rest fully switched off during off hours. The annual energy saving potential by avoiding standby for the entire department is the difference between the two results. To calculate the annual emissions due to electricity consumption of the anesthesia workstations in our department and the potential for GHG emission reduction by switching devices off instead of leaving them on standby, the electricity saving potential was multiplied by the respective emission factor. The annual electricity costs for the operation of one device was calculated by multiplying the price of electricity by the electricity consumption of the respective device and the number of hours of operation or standby mode per year. The annual cost-saving potential for the entire department by avoiding standby was calculated as annual power-saving potential multiplied by the price of electricity.

## Results

### Power consumption of anesthesia workstations

**Fig. 1:**
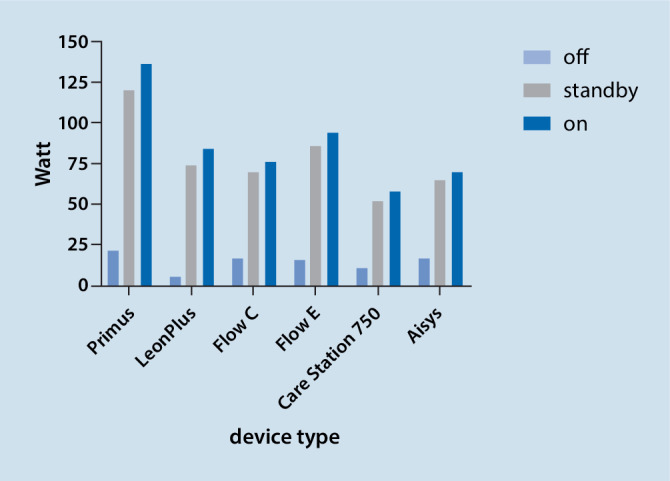
Electricity consumption (active power in watt) of different anesthesia workstations while switched off, in standby mode or fully on

When switched off the power consumption of the anesthesia workstations was in a range from 5 W (Löwenstein Leon Plus) to 22 W (Dräger Primus). In standby mode, active power was between 52 W (GE Care Station 750) and 120 W (Dräger Primus). While in use, anesthesia workstations consumed between 58 W (GE Care Station 750) and 136 W (Dräger Primus). The respective values for all devices are depicted in Fig. 1.

### Annual power consumption and electricity saving potential

**Fig. 2:**
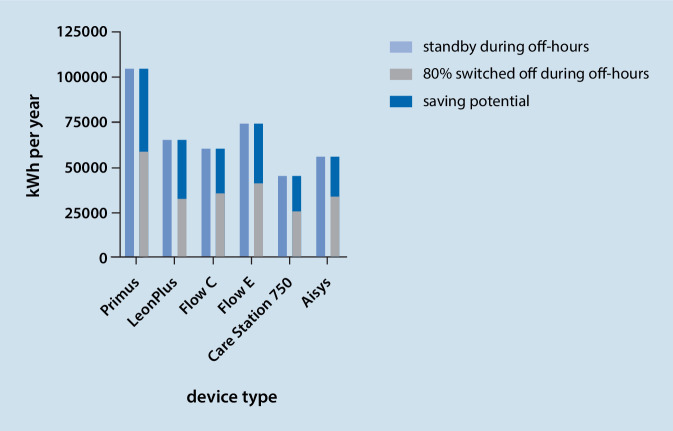
Annual electricity consumption in kilowatt-hours (kWh) for all 96 anesthesia workstations in our department with all devices either left on standby or 80% of devices switched off during off hours. The upper part of the stacked columns represents the electricity saving potential

We calculated 2527 h of regular operation and 6233 off hours per year (public holidays considered). Our department operates 96 anesthesia workstations. Assuming that approximately 80% of these workstations are not necessary for serving time-critical emergencies during off hours, 76 devices could safely be switched off instead of being left on standby during off hours and 20 would have to remain on standby. Figure 2 shows the annual electricity consumption for the entire department with either all devices in standby or 76 of 96 devices switched off and 20 of 96 devices on standby, plus the electricity saving that could be obtained by avoiding standby for the respective device type.

### Greenhouse gas emission reduction potential

The emission factor of the electricity provided to our hospital in 2022 was 0.427 kg CO_2_-eq per kWh. Emission factors in the European Union in 2022 ranged from 0.007 kg/kWh in Sweden to 0.666 kg/kWh in Poland, with an EU-wide average of 0.251 kg/kWh and nationwide average in Germany of 0.366 kg/kWh. The emission factor was 0.068 kg/kWh in France (2022), 0.207 kg/kWh in the UK (2023), and 0.389 in the USA (2022). The potential for GHG emission reduction is expressed by multiplying the electricity saving potential by the respective emission factor. The results for the reduction potential for the emission factor of our hospital and for the emission factors of the mentioned exemplary countries are listed in Table 1.

**Table 1:** Calculations of the annual emissions in tons of CO_2_-equivalents (t CO_2_-eq) due to the electricity consumption of all 96 devices in our department at different emission factors

	Annual emissions with all devices left in standby mode (t CO_2_-eq)							Annual emissions with 76 devices switched off and 20 in standby (t CO_2_-eq)							Annual emission saving potential by switching off 80% of devices during off hours (t CO_2_-eq)						
	UHC	GER	SW	PL	FRA	UK	USA	UHC	GER	SW	PL	FRA	UK	USA	UHC	GER	SW	PL	FRA	UK	USA
Primus	44.75	38.36	0.73	69.79	7.13	21.69	40.77	24.93	21.37	0.41	38.88	3.97	12.08	22.71	19.82	16.99	0.32	30.92	3.16	9.61	18.06
LeonPlus	27.61	23.66	0.45	43.06	4.40	13.38	25.15	13.65	11.70	0.22	21.29	2.17	6.62	12.44	13.96	11.96	0.23	21.77	2.22	6.77	12.71
Flow C	25.50	21.86	0.42	39.78	4.06	12.36	23.23	14.98	12.84	0.25	23.37	2.39	7.26	13.65	10.52	9.02	0.17	16.41	1.67	5.10	9.58
Flow E	31.71	27.18	0.52	49.46	5.05	15.37	28.89	17.55	15.04	0.29	27.38	2.80	8.51	15.99	14.16	12.14	0.23	22.08	2.25	6.86	12.90
Care Station 750	19.29	16.54	0.32	30.09	3.07	9.35	17.58	10.80	9.26	0.18	16.84	1.72	5.24	9.84	8.50	7.28	0.14	13.25	1.35	4.12	7.74
Aisys	23.86	20.45	0.39	37.21	3.80	11.57	21.74	14.15	12.13	0.23	22.07	2.25	6.86	12.89	9.71	8.32	0.16	15.14	1.55	4.71	8.84

*UHC* University Hospital of Cologne, *GER* Germany, *SW* Sweden, *PL* Poland, *FRA* France, *UK* United Kingdom, *USA* United States of America

*Left columns* emissions with all 96 devices left in standby during off hours. *Middle columns* emissions with 76 devices switched off and 20 left in standby mode during off hours. *Right columns* emission saving potential by switching off 80% (76/96) of devices during off hours

### Calculations for electricity costs and potential financial savings

According to the Federal Statistical Office of Germany, the average price of electricity for commercial consumers in Germany was 0.25 € per kWh in 2022. The annual electricity costs for one workstation (fully on during regular operation hours and in standby during off-hours) ranged from approximately 118 € to 273 €. The detailed results are listed in Table 2. Avoiding standby during off hours in roughly 80% of all our anesthesia workstations can save between 4974 € with GE Care Station 750 and 11,606 € with Dräger Primus of electricity costs per year. The results for all device types can be found in Table 2.

**Table 2:** Calculations of annual electricity cost at an electricity price of 0.25 € per kWh. Values for a single device, for all 96 devices when left in standby mode during off hours, for all devices when 76 are switched off and 20 left in standby mode during off hours and for the cost saving potential by switching off 80% (76/96) of devices during off hours. Amounts are presented in euros (€)

	Annual electricity costs per device, standby during off hours (€)	Annual electricity costs with all devices left in standby (€)	Annual electricity costs with 76 devices switched off and 20 in standby mode (€)	Annual cost saving potential by switching off 80% of devices during off hours (€)
Primus	273	26,199	14,594	11,606
LeonPlus	168	16,164	7993	8171
Flow C	156	14,931	8773	6158
Flow E	193	18,566	10,276	8290
Care Station 750	118	11,296	6323	4974
Aisys	146	13,969	8284	5684

## Discussion

Power consumption varies considerably between device types. The most energy-intensive device (Primus) consumed 134% more than the least energy-intensive device (Care Station 750) during operation. A common feature of all devices is the high power consumption during standby, 88–93% of the power needed during operation. This high percentage reveals potential to save electricity, GHG emissions and cost by switching devices off instead of leaving them on standby wherever feasible. In our hospital, between 8.5 and 19.8 tons of CO_2_-equivalent and between 4974 € and 11,606 € could be saved annually by avoiding standby during off hours in 80% of the anesthesia workstations. Electricity costs for the operation of one anesthesia workstation amount to 118 € to 273 € per year. The CO_2_-equivalent emitted per year ranges from 201 kg to 466 kg per device.

Our study confirmed previous analyzes demonstrating that scope 2 emissions from electricity consumption of medical devices represent only a small part of the total GHG emissions generated by anesthesia and surgery [3, 4, 20]. Especially with desflurane, scope 1 emissions from volatile anesthetics or nitrous oxide constitute the main share of GHG emissions from anesthesia [4, 7]. Scope 2 emissions in anesthesia and surgery are much more heterogeneous than scope 1 emissions. A considerable percentage of scope 2 emissions originate from infrastructural characteristics of operating theaters (e.g., heating, ventilation, lighting) and are used for anesthesia and surgery, whereas scope 1 emissions apply to anesthesia alone. In this analysis, we chose to investigate an item of scope 2 emissions that can be controlled by anesthesiologists themselves without external influences. As described above, the absolute electricity consumption of anesthesia workstations is modest compared with other sources of scope 2 emissions [3, 20]; however, it is noteworthy that the power consumption in standby mode is approximately 90% of that during an operation. As it appears to be common practice in many hospitals to leave machines on standby during prolonged times, if not constantly, this gives a leverage point to reduce electricity consumption and thereby to reduce GHG emissions and cost by simple behavioral changes. If approximately 80% (76/96) of the machines in our hospital were switched off instead of being left on standby during off hours, between 8.4 and 19.5 tons CO_2_-equivalent could be saved annually at the current emission factor. The carbon footprint of this intervention largely depends on the emission factor of the respective hospital. The emission factor largely depends on the sources of energy used for the generation of electricity. With the average emission factor in Sweden, the lowest in the European Union due to a high share of renewables and nuclear energy, the same power saving would result in a reduction of emissions of 0.14 to 0.32 tons, whereas with the average emission factor in Poland, the highest in the European Union due to a high percentage of coal combustion to generate electricity, it would imply a reduction of 13.2 tons to 30.9 tons. At the average emission factor in Germany, emissions could be reduced by 7.3 tons to 17.0 tons. Considering that the annual CO_2_-emissions per capita are 8.1 tons in Germany [21], the potential savings by avoiding standby in our hospital equals the emissions of one or two citizens in Germany. Compared with the impact of volatile anesthetics, which can create more than 1000 tons CO_2_-equivalent per year in a large hospital [3], this is a small potential for CO_2_ reductions. Interventions targeting at reducing the consumption of volatile anesthetics carry a much higher potential for reduction of GHG emissions: A recent study calculated a potential of a reduction of 1.8 kg CO_2_-equivalent (global warming potential 20 years, GWP_20_) due to saving of sevoflurane during a 45min case if modern anesthesia workstations were used most efficiently [9]. A clinical decision support tool aiding anesthesiologists to use volatile anesthetics more efficiently led to a saving of 4.1 ml desflurane or 3.8 ml per MAC‑h sevoflurane [22]. Depending on the estimation of the relative GWP of volatile anesthetics, this amounts to a reduction in CO_2_-equivalent (GWP_20_) of 22 kg to 41 kg for desflurane and 2.0 kg to 4.6 kg for sevoflurane [23–26]. Avoiding standby and switching the device off instead only leads to a reduction of emissions of less than 0.1 kg CO_2_-equivalent for one anesthesia workstation per hour even at a high emission factor. Hence, the impact of saving electricity by switching off medical devices is smaller than the impact of reducing the consumption of volatile anesthetics. Also compared with other electricity consumers in the operating room, such as forced air warming blanket devices or anesthetic gas scavenging systems [27], the saving potential appears modest; however, this easily feasible measure to reduce emissions should not be left unused.

The electricity costs of anesthesia workstations are between 118 € and 273 € per device per year for 2527 h of operation and standby mode during the remaining 6233 h. For the 96 devices in our department, this amounts to 11,296–26,199 € per year, provided that the relatively low price for commercial consumers remains. By avoiding standby in 80% of all devices during off hours, approximately 40–45%, or 5000–11,000€ of electricity costs could be saved annually. In the light of the total budget of a large clinical department, the financial savings that could be reached by this intervention are humble but easily achievable. From a merely economical perspective, different electricity consumption does not seem to play a major role in considerations of different types of anesthesia workstations.

## Limitations

Some limitations must be considered: First, we investigated the electricity consumption of the anesthesia workstations during clinical use. To fully analyze the ecological profile of different devices, features during manufacturing, transport, consumption of air and oxygen during use, etc. also need to be considered. This would require a complete life cycle assessment of the respective device. Also, given that the age of the machines studied was heterogeneous, battery charging behavior might differ, which could potentially influence electricity consumption while switched off. In order to minimize this potential bias all devices were connected to the AC mains to fully precharge the batteries. In future investigations longer (e.g. 24 h) measurement periods might help to gain a more detailed profile of the battery charging profiles of the respective devices. Second, the calculations are partly based on assumptions. For example, it is uncertain which percentage of standby avoidance could be achieved in the real world. This could be studied by measuring power consumption before and after an educational measures encouraging personnel to switch off devices when not in use. Third, the GHGs emitted and the costs caused by the electricity consumption of anesthesia workstations depend on the respective emission factor and the price of electricity. To give an overview of the range of emissions and potential savings, we have presented calculations for different (local and national) emission factors. Actual values could vary for every hospital and every time point in an annual and circadian rhythm. Given that the emission factor of electricity in Germany is higher during the night most of the year [28], switching off devices and thereby saving power during the night might in fact lead to higher emission savings than calculated with the annual values.

## Conclusion

We have shown that power consumption varies considerably between different anesthesia workstations. All devices exhibit a high power consumption in standby mode, approximately 90% of that during operation. Avoiding standby mode during off hours can save energy. The reductions in GHG emissions and electricity costs that can be reached with this intervention in a large anesthesiology department are between 8.5 and 19.8 tons of CO_2_-equivalent and 4974 € and 11,606 €. These savings depend largely on the respective emission factor and price of electricity. Compared with other sources of GHG emissions in anesthesiology, particularly volatile anesthetics, the potential for emission reductions by optimizing electricity use of anesthesia workstations remains small; however, we think that every feasible opportunity to decrease the carbon footprint of our clinical work should be seized. Additionally, manufacturers should be encouraged to develop technical solutions to reduce the electricity demand in standby mode.

## Declarations

Ethical approval was not deemed necessary by the managing director of the ethics committee at the medical faculty of the University of Cologne.
